# C-Reactive Protein as Predictive Biomarker for Response to Chemoradiotherapy in Patients with Locally Advanced Rectal Cancer: A Retrospective Study

**DOI:** 10.3390/cancers14030491

**Published:** 2022-01-19

**Authors:** Fátima Aires, Darlene Rodrigues, María Piñeiro Lamas, Maria Teresa Herdeiro, Adolfo Figueiras, Maria José Oliveira, Margarida Marques, Ana Teresa Pinto

**Affiliations:** 1 Radiotherapy Department of Centro Hospitalar Universitário de São João (CHUSJ), 4200-319 Porto, Portugal; darlenerodrigues.dr@outlook.pt (D.R.); margarida.reis@chsj.min-saude.pt (M.M.); 2 ICBAS–Instituto de Ciências Biomédicas Abel Salazar, University of Porto, 4050-313 Porto, Portugal; mariajo@ineb.up.pt; 3 CINTESIS–Center for Health Technology and Services Research, University of Porto, 4200-450 Porto, Portugal; 4 Consortium for Biomedical Research in Epidemiology and Public Health (CIBER Epidemiology and Public Health–CIBERESP), 15706 Santiago de Compostela, Spain; maria.pineiro@usc.es (M.P.L.); adolfo.figueiras@usc.es (A.F.); 5 Health Research Institute of Santiago de Compostela (IDIS), University of Santiago de Compostela, 15706 Santiago de Compostela, Spain; 6 Department of Medical Sciences, Institute of Biomedicine–iBiMED, University of Aveiro, 3810-193 Aveiro, Portugal; teresaherdeiro@ua.pt (M.T.H.); anapinto@ua.pt (A.T.P.); 7 Faculty of Medicine, University of Santiago de Compostela, 15706 Santiago de Compostela, Spain; 8 i3S–Instituto de Investigação e Inovação em Saúde, University of Porto, 4200-135 Porto, Portugal; 9 INEB–Instituto de Engenharia Biomédica, University of Porto, 4200-135 Porto, Portugal

**Keywords:** rectal cancer, neoadjuvant chemoradiotherapy, biomarkers

## Abstract

**Simple Summary:**

Most patients with locally advanced rectal cancer present resistance or a moderate response to neoadjuvant chemoradiotherapy (nCRT), which is considered the standard of care. To select patients who could benefit from nCRT, while avoiding unnecessary treatment-induced toxicity and surgery-associated morbidity, it is urgent to find biomarkers of response to chemoradiotherapy. Therefore, the aim of our retrospective study was to assess the potential of classical blood analytes collected before chemoradiotherapy as biomarkers of response to treatment and prognostics in locally advanced rectal cancer. Our results identified C-reactive protein ≤3.5 as a strong independent predictor of response to treatment and an independent predictor of disease-free survival (DFS) and overall survival (OS). Additionally, platelets were found to be independent predictors of DFS and OS and hemoglobin of DFS. These data might contribute to the personalization of rectal cancer treatment by guiding clinicians in decision-making regarding the best treatment strategy for each patient.

**Abstract:**

The standard of care for the treatment of locally advanced rectal cancer is neoadjuvant chemoradiotherapy (nCRT) followed by surgery, but complete response rates are reduced. To find predictive biomarkers of response to therapy, we conducted a retrospective study evaluating blood biomarkers before nCRT. Hemoglobin (Hg), C-reactive protein (CRP), platelets, carcinoembryonic antigen, carbohydrate antigen 19.9 levels, and neutrophil/lymphocyte ratio were obtained from 171 rectal cancer patients before nCRT. Patients were classified as responders (Ryan 0–1; ycT0N0), 59.6% (*n* = 102), or nonresponders (Ryan 2–3), 40.3% (*n* = 69), in accordance with the Ryan classification. A logistic regression using prognostic pretreatment factors identified CRP ≤ 3.5 (OR = 0.05; 95%CI: 0.01–0.21) as a strong independent predictor of response to treatment. Multivariate analysis showed that CRP was an independent predictor of disease-free survival (DFS) (HR = 5.48; 95%CI: 1.54–19.48) and overall survival (HR = 6.10; 95%CI 1.27–29.33) in patients treated with nCRT. Platelets were an independent predictor of DFS (HR = 3.068; 95%CI: 1.29–7.30) and OS (HR= 4.65; 95%CI: 1.66–13.05) and Hg was revealed to be an independent predictor of DFS (HR = 0.37; 95%CI: 0.15–0.90) in rectal cancer patients treated with nCRT. The lower expression of CRP is independently associated with an improved response to nCRT, DFS, and OS.

## 1. Introduction

Rectal cancer is one of the most common types of cancer [1]. Over the past few decades, the management of rectal cancer has significantly evolved, but neoadjuvant therapy, including radiotherapy and chemotherapy, has always been an indispensable part of the treatment. Particularly, in locally advanced rectal cancer (T3/T4 or node-positive rectal adenocarcinoma), preoperative chemoradiotherapy (nCRT), is the standard of care, aiming to reduce local recurrence, downstage, and downsize the tumor prior the potential radical surgery [2,3]. Currently, two standard preoperative therapy options are considered: short-course radiotherapy (25 Gy in five fractions over one week) with immediate or delayed surgery, and long-course chemoradiotherapy (45–50 Gy in 25 fractions over five weeks) with concurrent chemotherapy (most commonly 5-FU) and surgical treatment after 6–10 weeks [4,5].

Clinically, the response to nCRT is usually evaluated using endoscopy and imaging studies, like magnetic resonance imaging (MRI) and positron emission tomography (PET), which allow restaging, despite their limited accuracy for determining T-stage and lymph node involvement [6,7,8,9,10]. To properly determine both cancer staging and treatment response evaluation, the accurate pathological assessment of the surgical specimen is essential. It is usually performed following the guidelines of Ryan [11], which consider the following classification of the surgical specimen: no viable cancer cells (Ryan 0), single cells/small groups of cancer cells (Ryan 1), residual cancer outgrown by fibrosis (Ryan 2), and extensive residual cancer (Ryan 3). Importantly, only 10–30% of the patients exhibit a complete pathological response [12,13,14], while about 45% present a partial response, and the remaining are resistant [15,16,17,18]. Patients with a complete pathological response to nCRT have lower rates of local recurrence and improved survival as compared to patients who did not achieve a complete pathological response [19]. This variability in response to nCRT in rectal cancer patients highlights the urgent need to find biomarkers able to predict the response to nCRT, by differentiating responsive from nonresponsive patients before nCRT. This would avoid unnecessary chemoradiotherapy-associated toxicity in patients who will not achieve a complete pathological response, and at the same time, it would save many human, logistic and financing resources.

The predictive value of many molecular biomarkers in biopsy and surgical specimens has been investigated [6], but none can predict the therapeutic effect of nCRT. On the other hand, blood samples are a very interesting source of biomarkers since they are easily collected, allowing noninvasive monitoring at several time points. The carcinoembryonic antigen (CEA) is the recommended biomarker for colorectal cancer monitoring [20,21,22]. Its potential as a predictive biomarker of response to nCRT has been investigated, while its prognostic value alone has been controversial [23,24,25,26]. By considering CEA together with a carbohydrate antigen (CA) 19.9, Zheng recently showed that the normalization of elevated CEA + CA 19.9 levels by nCRT was an independent prognostic protective factor in patients with locally advanced rectal cancer [27]. Focusing on hematological parameters routinely evaluated before nCRT, low levels of hemoglobin (anemia) have been associated with less tumor regression [28], while elevated platelet count (thrombocytosis) seems to predict poor response [29]. Regarding inflammation, which is particularly relevant as a hallmark of cancer [30], an elevated pre-nCRT neutrophil-to-lymphocyte ratio (NLR) is associated with poor pathological response and prognosis [31,32,33]. The predictive value of other molecules, nonroutinely analyzed, has also been investigated [34,35,36].

Despite the growing interest on predictive biomarkers for radiotherapy response, none has yet reached the clinic, and external validation using larger cohorts is still required. Aiming to improve the clinical evidence of new biomarkers of response to nCRT and strengthen data on other promising ones, we developed a retrospective study, involving 171 rectal cancer patients, from whom blood was collected before nCRT. The predictive and prognostic value of some routinely analyzed blood molecules were evaluated: CEA and CA 19.9 (commonly used cancer biomarkers), hemoglobin and platelets (promising hematological biomarkers in rectal cancer), and NLR and C-reactive protein (CRP) (systemic inflammatory response markers).

In the end, we expect to contribute to finding accurate and inexpensive predictors of response to nCRT which can be easily accessible in clinical practice. Hopefully, these will allow clinicians to select patients who can benefit from nCRT, thereby avoiding overtreatment-associated toxicity and unnecessary invasive procedures, while improving patients’ quality of life and saving costs.

## 2. Materials and Methods

### 2.1. Patients

From January 2013 to December 2019, 171 rectal cancer patients were treated with long-course nCRT at our hospital, Centro Hospitalar Universitário de São João (CHUSJ, Porto). Indications for neoadjuvant treatment include rectal adenocarcinoma stages cT3–4, N any; any T, N1–2, M0 and patients with high risk or rejecting radical surgery for whom a multidisciplinary meeting recommends preoperative CRT. Patients with contraindications to chemotherapy, who received short-course radiotherapy (these are usually the patients with more comorbidities in our institution), or who did not complete the neoadjuvant treatment were excluded. Clinical staging, before and after neoadjuvant treatment, was done by thoracoabdominal computer tomography scan and pelvic resonance. Histological examinations were carried out according to the American Joint Committee on Cancer system. For all recruited patients there were available data regarding Hg, platelets, CEA, CA 19.9, NLR, and CRP levels before nCRT. The study was approved by the Ethical Committee of our hospital (no. 205/19).

### 2.2. Chemoradiotherapy

All patients received pelvic radiation in accordance with international guidelines [37,38]. Radiotherapy was delivered as three-dimensional conformal radiotherapy (3DCRT) or Volumetric Modulated Arc Therapy (VMAT) to a total dose of 45–55 Gy. Most patients received concomitant CRT with administration of capecitabine, an oral prodrug of 5-fluorouracil (5-FU) (*n* = 157; 94.1%). The other chemotherapy regimens included capecitabine + FOLFOX, CAPOX, or FOLFOX. Blood samples were collected before nCRT, from which data regarding Hg, CRP, platelets, NLR, CEA, and CA 19.9 were obtained and analyzed.

### 2.3. Treatment Response

Pathological response to treatment was evaluated histologically 8–10 weeks after nCRT on surgical resections, following the Ryan score for tumor regression [11]: no viable cancer cells (Ryan 0), single cells/small groups of cancer cells (Ryan 1), residual cancer outgrown by fibrosis (Ryan 2), and extensive residual cancer (Ryan 3). Accordingly, patients were classified into two groups: responders (Ryan 0–1; complete or moderate tumor regression) and nonresponders (Ryan 2–3; minimal or no regression). Clinical total responders (ycT0N0) followed the “watch and wait” strategy, maintaining the surveillance and were included in the responder group. Patients with irresectable tumors were included in the nonresponder group.

### 2.4. Clinical End Points

Overall Survival (OS) was defined as the interval between the end of nCRT and the occurrence of death, whatever the cause. Disease-free survival (DFS) was defined as the interval between the end of nCRT and the occurrence of the first observed oncologic event, such as local or metastatic recurrence, second cancer, or death from any cause. Patients without events at the time of analysis were censored on the date of the last informative follow-up.

### 2.5. Statistical Analysis

All analyses were performed using the free R statistical software environment [39]. A receiver operating characteristic (ROC) curve was used to determine the cutoff values of Hg (12.2 g/dL), CRP (3.5 mg/L), platelets (253.5 × 10^9^/L), NLR (2.3), CEA (2.7 ng/mL), and CA 19.9 (3.5 U/mL) before neoadjuvant treatment. ROC analysis and the area under the ROC curve (AUC) for all the mentioned blood parameters were used to verify the predictive capability of the responders to nCRT in our series (Figure S1). The AUC confidence intervals were computed with Delong’s method [40] and the optimal cut-off was obtained with de Youden’s J statistic [41]. For the comparison of the groups calculated by the threshold predictor value, the Chi-square test was used. Logistic regression was performed to determine univariate relationships between pretreatment clinical predictors and response to nCRT. Bivariate analysis was performed to select independent variables with a *p*-value < 0.2. The selected predictors were studied in multivariate analysis and those that had greater statistical significance were successively eliminated on the condition that the coefficients of the main exposure variables did not change by more than 10% and that the Schwartz’s Bayesian Information Criterion (BIC) improved. The final predictive multivariate model was internally validated [42,43] with the repeated data-splitting technique [44]. According to this approach, a portion of the sample (75%) was randomly selected (training sample) for model development and tested on the remaining 25% (testing sample). This procedure was repeated 1000 times to get different samples at each repetition to examine different scenarios. OS and DFS were analyzed using the Kaplan–Meier method, and differences were examined using log-rank tests. Cox proportional hazards regression modelling was used to assess the prognosis, adjusted for significant clinical covariates.

## 3. Results

### 3.1. Clinicopathologic Characteristics

From January 2013 to December 2019, 171 rectal cancer patients were treated with long-course nCRT in our hospital center. Patient´s demographics and tumor characteristics are presented in Table 1. The mean age of the studied cohort was 62 years (31–84). Most patients were male (*n* = 108; 63.2%). A very good performance status (ECOG = 0) was predominant (*n* = 138; 80.7%). At initial presentation, most patients presented a medium or inferior rectal tumor (*n* = 141; 82.4%) classified as cT3 (*n* = 121; 70.3%) and were node (cN) positive (*n* = 136; 79.5%). Total radiotherapy dose varied between 45–55 Gy, being applied in most cases through 3D-CRT technique (*n* = 147; 86%) or VMAT in the remaining patients (*n* = 24; 14.0%). As concomitant chemotherapy, most patients received 5-FU orally (capecitabine) (*n* = 157; 91.8%) or intravenously (*n* = 4; 2.3%). Response to treatment is listed in Table 2. Thirteen patients (7.6%) developed a complete clinical response (ycT0N0), 33 (19.3%) developed a complete pathologic response (pT0N0, Ryan 0), and 56 (32.7%) presented a moderate response, according to Ryan´s Tumor Regression Grade Scoring System (Ryan 1). All these patients were included in the responder group (*n* = 102; 59.6%). The nonresponder group (*n* = 69, 40.3%) included patients with tumors classified as Ryan 2–3 (*n* = 64; 37.4%) or unresectable tumors (*n* = 5; 2.9%).

### 3.2. Relationships between Pretreatment Clinicopathologic Factors and Response to Treatment

The multivariate regression logistic analysis using prognostic pretreatment factors identified CRP ≤ 3.5 mg/L (OR 0.05; 95% CI 0.007–0.212) as a strong independent predictor of response to treatment (Table 3). This was confirmed with the internal validation of the model (Table 4).

**Figure 1:**
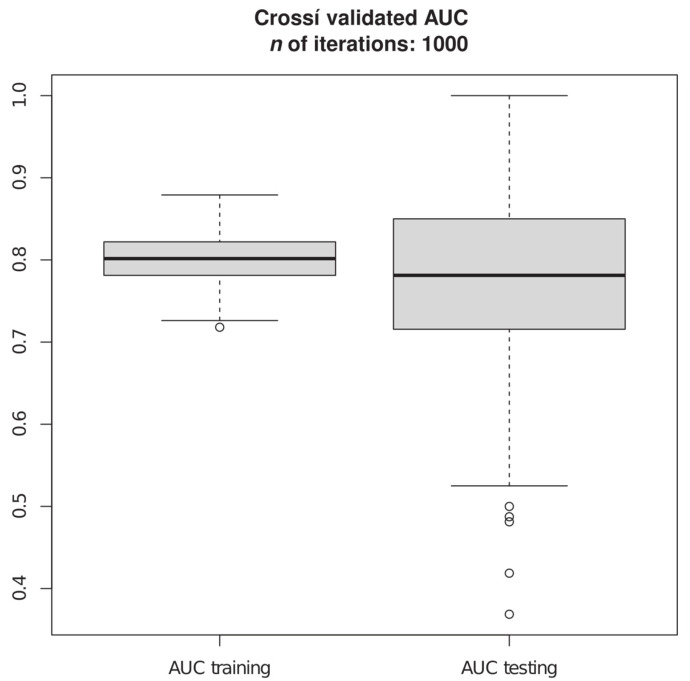
Internal validation: training versus testing distributions.

The model fitted on the full sample has an excellent discrimination (C = 0.81; see Figure S2). The training distribution of the C statistic is also very good. The median is 0.81 and the distribution is concentrated around the median (IQR/2 = 0.02; C Var = 2.3%). Furthermore, the minimum value of the distribution is 0.74, which is still an acceptable value for discrimination. Thus, the model discriminates very well between the two classes of outcome in the 1000 training samples. The validation distribution of the C statistic proves to be an acceptable model of the validation samples. The median value of the distribution is 0.80 (Figure 1).

Considering the “Watch&Wait” as a very important subgroup of patients, who present a complete clinical response after nCRT without requiring surgery, we compared CRP levels in this subgroup (*n* = 13) vs. the nonresponder group (*n* = 69). A resulting OR of 0.069 (0.007–0.463) and a *p*-value of 0.0086 were obtained, highlighting the contribution of this subgroup, even with a small number of patients, for CRP as a strong independent predictor of response to treatment.

Of note, an additional statistical analysis, excluding the different minority chemotherapy regimens that existed in our study, still demonstrates CRP as the only significative factor at multivariate analysis (see Table S1), evidence that they do not constitute a bias in the study design.

### 3.3. Relationships between Pretreatment Clinicopathologic Factors and Prognosis

The median follow-up was 34 months, during which 5 patients presented local recurrence, 21 patients presented metastatic disease and 36 deaths were reported. Particularly, regarding the “Watch&Wait” subgroup, none of the 13 patients presented local regrowth. OS and DFS classified based on cT, ECOG, Hg, platelets, CRP, NLR, CEA, and Ca19.9 prior to nCRT are shown in Table 5.

**Figure 2:**
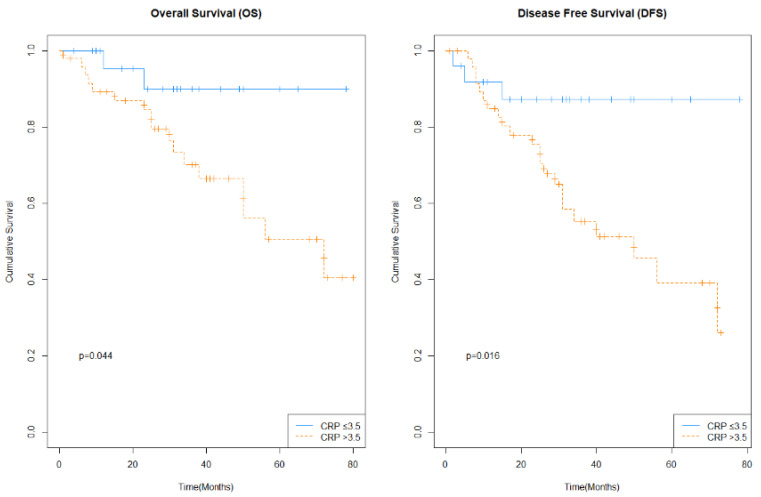
Kaplan–Meier curves for overall survival (OS) and disease-free-survival (DFS) for C-reactive protein (CRP), prior to neoadjuvant chemoradiotherapy (nCRT)) in patients with locally advanced rectal cancer.

Multivariate analysis using a Cox proportional hazards model showed that CRP was an independent predictor of DFS (Hazard Ratio (HR) = 5.481; 95% CI 1.542–19.485) and OS (HR = 6.096; 95% CI 1.267–29.323) in patients with rectal cancer treated with nCRT. Kaplan–Meier curves for OS and DFS for CRP, prior to chemoradiotherapy in patients with rectal cancer are shown in Figure 2.

The multivariate analysis also showed that platelets were an independent predictor of DFS (HR = 3.068; 95% CI 1.29–7.296) and OS (HR = 4.654; 95% CI 1.659–13.053), and Hg was revealed to be an independent predictor of DFS (1/HR = 2.7; 95% CI 1.10–6.62) in patients with rectal cancer treated with nCRT. The Kaplan–Meier curves for OS and DFS for Hg and platelets prior to chemoradiotherapy in patients with rectal cancer are shown in the supplementary material (Figure S3).

## 4. Discussion

The present retrospective study evaluated the association between blood analytes obtained before nCRT and the response to treatment of patients with locally advanced rectal cancer. Two groups were considered: responder (Ryan 0–1) vs. nonresponder (Ryan 2–3), according to Ryan guidelines. Although different grading systems [11,45,46,47,48] are used in the literature, the Ryan [11] system is the one recommended by the American Joint Committee on Cancer TNM Staging Classification for Rectal Cancer (8th ed, 2017) to grade tumor response [49]. As reported before [15], in our study the response to nCRT was also variable; a complete clinical or pathologic response was achieved in 26.9% of patients, a moderate response in 32.7%, and no response in 40.3%.

We demonstrated that low CRP levels before nCRT predict a good response to treatment and anticipate DFS and OS. As far as we know, this is the first time that CRP *per se* is shown to be independently associated with response to treatment. Plasma CRP has been proposed as a sensitive serological surrogate parameter for elevated levels of proinflammatory cytokines stimulating angiogenesis, tumor proliferation, and growth, being an easily measurable biomarker, which is routinely analyzed before treatment initiation [50,51].

Unlike our results, Buijsen, who investigated various pretreatment biomarkers as predictive factors for tumor response after nCRT in rectal cancer patients, did not detect a significant association between the pretreatment CRP level and tumor response after nCRT. Of note, the authors used ypT0–2N0 as the definition for responder and all other ypTN stages for nonresponder patients [52]. Dreyer et al. also examined the association between systemic inflammation and nCRT in patients with rectal cancer, having dichotomized patients as good responders (tumor response grade (Rodel’s TRG) 3 and 4) or as poor or no responders (Rodel’s TRG 0, 1, and 2). Serum measurements of hemoglobin, differential white cell counts, CRP, albumin, and modified Glasgow prognostic score (mGPS), which is a combination of CRP and albumin levels, were obtained before and after nCRT. The authors showed that a high systemic inflammatory response before nCRT, given by higher mGPS, was associated with a poor pathologic response, as quantified by the tumor regression grade [53].

Regarding the prognostic value of CRP, a systematic literature review determined the relationship between elevated CRP and prognosis in people with solid tumors in 90% of the cases, which was particularly notable in gastrointestinal malignancies [54]. Similar to our results, an elevated CRP also predicted a bad prognosis. Particularly, in locally advanced rectal cancer patients, Parti et al. [55] investigated the association of the pretreatment CRP plasma level with survival outcomes in a cohort of 423 consecutive patients treated with nCRT. In a multivariate analysis, the pretreatment CRP remained a significant prognostic factor for recurrence-free survival (HR = 1.013, 95%CI 1.001–1.025; *p* = 0.036), loco-regional control (HR = 1.014, 95% CI 1.001–1.027; *p* = 0.031), and metastases-free survival (HR = 1.013, 95% CI 1.000–1.027; *p* = 0.046). The results support the hypothesis that an elevated pretreatment CRP level is a predictor of poor outcome. Toiyama et al. [56] also analyzed the prognostic impact of the pretreatment CRP level in a cohort of 84 patients treated with nCRT and subsequent total mesorectal excision. The authors identified an elevated pretreatment CRP level as a significant prognostic factor for poor OS and DFS, in line with our results. Recently, the low levels of the lymphocyte–CRP ratio found in rectal cancer patients before nCRT were considered an independent prognostic factor for both recurrence-free survival and OS [57,58].

Although the CRP seems to have a predictive and prognostic value in rectal cancer patients treated with nCRT, it is a nonspecific marker of inflammation and might be influenced by several conditions, such as bacterial or viral infection, inflammatory diseases, connective tissue disorders, and medical treatments, although none of these conditions were identified in our cohort. Thus, a better understanding of how the initial patient inflammation status may help to predict the response to nCRT is still required. That could be determined, for instance, through the evaluation of the expression of several cytokines or polymorphisms in inflammatory genes.

Our study also showed that platelets were an independent predictor of DFS and of OS in patients with rectal cancer treated with nCRT, and Hg was revealed to be an independent predictor of DFS without an association to treatment response.

Toiyama [59] also found a significant association between elevated platelets and poor OS in patients with locally advanced rectal cancer who underwent neoadjuvant CRT, significantly predicting poor DFS. In fact, increased platelet count may indicate poor prognosis in other cancer types [60] also.

Regarding low Hg levels (anemia) in patients with diagnosed rectal cancer, it may indicate a larger tumor with advanced disease or an inherent feature of biologically aggressive behavior. It has been shown to be of prognostic value in patients with curable rectal cancer [61,62]. Khan [63] investigated whether pretreatment Hg levels act as a biomarker in the management of patients with locally advanced rectal cancer. They found that local recurrence was more common in patients with a pretreatment Hb of <12 g/dL (HR = 1.78) over a median follow up of 24 months, but this was not statistically significant (*p* = 0.08).

Although the literature reports several biomarkers capable of predicting response to neoadjuvant therapy in rectal cancer, none have been introduced into the routine practice due to problems with methodology and validation [64]. Thus, to find clinically relevant biomarkers of response to nCRT in rectal cancer patients, reinforce the results here presented, and better understand how CRP levels correlate with patient initial inflammatory status, we are now conducting a similar, but prospective, clinical study. This is intended to overcome the limitations of the present retrospective study by (i) collecting biomarker data before and after treatment, clarifying whether, for instance, a reduction of CRP levels after treatment may improve the outcome and (ii) using a larger cohort of patients, including a validation cohort, which will be determinant to validate the cut-off levels presented.

## 5. Conclusions

Our findings demonstrated that the lower expression of CRP levels seem to be independently associated with response and prognosis to treatment in patients with locally advanced rectal cancer who underwent long course nCRT. Determination of the pretreatment CRP level could provide additional prognostic information and contribute to the identification of patients who might be candidates for a more aggressive local or systemic treatment approach. Nevertheless, the analysis of this parameter should not be the only one to be taken into consideration for the prediction of the response in rectal cancer and cannot be considered as a definitive result, but rather, as a statement of hypothesis for further prospective studies. In addition, the classification of response to neoadjuvant therapy through different grading systems, using either clinical or pathology criteria, should be considered during the study design. 

## Figures and Tables

**Table 1:** Clinicopathologic characteristics of the 171 patients included in the study.

Factors	*n* (%)	Factors	*n* (%)
Age (years)		Clinical nodal stage (cN)	
		Positive	136 (79.5)
Mean (min-max)	61.77 (31–84)	Negative	35 (20.5)
Gender		TNM stage	
		1	1 (0.6)
Male	108 (63.2)	2	32 (18.7)
Female	63 (36.8)	3	138 (80.7)
ECOG		Neoadjuvant Radiotherapy	
0	138 (80.7)	3DCRT	147 (86.0)
1	32 (18.7)	VMAT	24 (14.0)
2	1 (0.6)	Median Dose (Gy) (min-max)	50 (45–55)
Tumor localization		Neoadjuvant chemotherapy regimen	
Superior	30 (17.5)	Capecitabine	157 (91.8)
Medium	71 (41.5)	5-FU	4 (2.3)
Inferior	70 (40.9)	Other	10 (5.9)
Clinical tumor stage (cT)		Clinical nodal stage (cN)	
2	12 (7.0)		
3	121 (70.3)	Positive	136 (79.5)
4	38 (22.1)	Negative	35 (20.5)

ECOG: Eastern Cooperative Oncology Group Performance Status; TNM: tumor node metastasis; 5-FU: 5-fluorouracil; 3DCRT: three-dimensional conformal radiation therapy; VMAT: volumetric modulated arch therapy; min: minimum value; max: maximum value.

**Table 2:** Characteristics of responder and nonresponder groups.

Patient Groups		*n* (%)
Responders	Complete clinical response (Watch&Wait: ycT0N0M0)	13 (7.6)
	Complete pathologic response (ypT0N0M0)–Ryan 0	33 (19.3)
	Moderate response–Ryan 1	56 (32.7)
	**Total**	**102 (59.6)**
Nonresponders	Ryan 2	43 (25.1)
	Ryan 3	21 (12.3)
	Unresectable	5 (2.9)
	**Total**	**69 (40.3)**

Ryan: Ryan Tumor regression grade.

**Table 3:** Comparison between pretreatment clinical factors and response to neoadjuvant chemoradiotherapy (nCRT). Odds ratios and 95% confidence intervals are generated from the logistic regression model.

Factors	Univariate Analysis OR (95% IC)	*p*	Multivariate Analysis OR (95% IC)	*p*
cT				
2–3	1 (-)			
4	0.457 (0.218–0.946)	0.0358		
ECOG				
0	1 (-)			
1–2	1.051 (0.487–2.328)	0.9007		
Hg (g/dL) *				
≤12.2	1 (-)			
>12.2	2.808 (1.412–5.692)	0.0036		
CRP (mg/L) *				
≤3.5	1 (-)			
>3.5	0.057 (0.009–0.223)	<0.0001	0.05 (0.007–0.212)	<0.0001
Platelets (×109/L) *				
≤253.5	1 (-)			
>253.5	0.354 (0.183–0.677)	0.0018		
NLR *				
≤2.3	1 (-)			
>2.3	0.725 (0.39–1.339)	0.306	2.181 (0.661–8.234)	0.2181
CEA (ng/mL) *				
≤2.7	1 (-)			
>2.7	0.457 (0.231–0.88)	0.0213	0.358 (0.081–1.387)	0.148
CA 19.9 (U/mL) *				
≤3.5	1 (-)			
>3.5	0.58 (0.295–1.113)	0.1062		

OR: odds ratio; CI: confidence interval; CT: clinical tumor; ECOG: Eastern Cooperative Oncology Group Performance Status; CPR: C-reactive protein; NLR: ratio neutrophil lymphocyte, CEA: carcinoembryonic antigen; CA 19.9: carbohydrate antigen 19.9. * Cut offs–ROC curve analysis.

**Table 4:** Internal validation results.

Discrimination: C Statistic						
	FIT = 100%	FIT = 75%—1000 iterations				
	FULL	MEDIANE	IQR/2 *	C Var **	Min	Max
C training	0.81	0.81	0.02	2.30	0.74	0.91
C testing		0.80	0.06	7.43	0.37	1.00

* Half of the interquartile range; ** ratio between IQR/2 and the median.

**Table 5:** Results of bivariate and multivariate Cox regression analysis for overall survival (OS) and disease-free survival (DFS). Multivariate analysis was performed with variables that showed statistical significance < 0.2 by the univariate analysis; subsequently, the variables with higher levels of statistical significance were eliminated from this model on the condition that the coefficients of the main exposure variables did not change by more than 10% and that the BIC improved.

Factors	OS						DFS					
	Univariate Analysis HR (95% IC)	Univariate Analysis 1/HR (95% IC)	*p*	Multivariate Analysis HR (95% IC)	Multivariate Analysis 1/HR (95% IC)	*p*	Univariate Analysis HR (95% IC)	Univariate Analysis 1/HR (95% IC)	*p*	Multivariate Analysis HR (95% IC)	Multivariate Analysis 1/HR (95% IC)	*p*
cT			0.0021	--		--			0.0031	--		--
2–3	1 (-)						1 (-)					
4	2.901 (1.47–5.728)	0.344 (0.17–0.68)					2.443 (1.352–4.414)	0.409 (0.22–0.74)				
ECOG			0.3032	--		--			0.4619	--		--
0	1 (-)						1 (-)					
1–2	1.558 (0.67–3.623)	0.642 (0.28–1.49)					1.316 (0.633–2.734)	0.759 (0.37–1.58)				
Hg (g/dL) *			<0.001			0.0595			<0.001			0.0293
≤12.2	1 (-)			1 (-)			1 (-)			1 (-)		
>12.2	0.234 (0.118–0.464)	4.274 (2.16–8.47)		0.39 (0.146–1.038)	2.564 (0.96–6.85)		0.203 (0.113–0.366)	4.926 (2.73–8.85)		0.37 (0.151–0.905)	2.70 (1.10–6.62)	
CRP (mg/L) *			0.0633			0.0241			0.0259			0.0086
≤3.5	1 (-)			1 (-)			1 (-)			1 (-)		
>3.5	4.021 (0.926–17.459)	0.249 (0.06–1.08)		6.096 (1.267–29.323)	0.164 (0.03–0.79)		3.945 (1.179–13.203)	0.253 (0.07–0.85)		5.481 (1.542–19.485)	0.182 (0.05–0.65)	
Platelets (×10^9^/L) *			0.0026			0.0035			0.0001			0.0112
≤253.5	1 (-)			1 (-)			1 (-)			1 (-)		
>253.5	2.793 (1.433–5.444)	0.358 (0.18–0.69)		4.654 (1.659–13.053)	0.215 (0.07–0.60)		3.029 (1.722–5.327)	0.330 (0.18–0.58)		3.068 (1.29–7.296)	0.326 (0.14–0.77)	
NLR *			0.0012	--		--			0.0011	--		--
≤2.3	1 (-)						1 (-)					
>2.3	3.695 (1.674–8.158)	0.271 (0.12–0.59)					2.821 (1.514–5.255)	0.354 (0.19–0.66)				
CEA (ng/mL) *			0.1266			0.0918			0.018	--		--
≤2.7	1 (-)			1 (-)			1 (-)					
>2.7	1.808 (0.846–3.865)	0.553 (0.25–1.18)		5.818 (0.751–45.055)	0.172 (0.02–1.33)		2.255 (1.15–4.421)	0.443 (0.22–0.87)				
CA 19.9 (U/mL) *			0.6607	--		--			0.695	--		--
≤3.5	1 (-)						1 (-)					
>3.5	0.859 (0.436–1.693)	1.164 (0.59–2.29)					0.891 (0.501–1.585)	1.122 (0.63–1.99)				

HR: hazard ratio; CI: confidence interval; cT: clinical tumor; ECOG: Eastern Cooperative Oncology Group Performance Status; CPR: C-reactive protein; NLR: ratio neutrophil lymphocyte, CEA: carcinoembryonic antigen; CA 19.9: carbohydrate antigen 19.9. * Cut offs–ROC curve analysis.

## Data Availability

The data presented in this study are not available on request from the corresponding author. Due to the General Data Protection Regulation, the data presented in this research are not publicly available.

## Supplementary Materials

The following supporting information can be downloaded at: https://www.mdpi.com/article/10.3390/cancers14030491/s1, Figure S1: ROC curves for Hg (hemoglobin, g/dL), CRP (C-reactive protein, mg/L), platelets (x10^9^/L), NLR (ratio neutrophil lymphocyte), CEA (carcinoembryonic antigen, ng/mL), and CA 19.9 (carbohydrate antigen 19.9, U/mL) levels in patients in the nonresponders’ group versus responders’ group to nCRT, Figure S2: ROC curves for the multivariate regression logistic model fitted on the full sample, Figure S3: Kaplan–Meier curves for overall survival (OS) and disease-free-survival (DFS) for hemoglobin (Hg) and platelets prior to neoadjuvant chemoradiotherapy (nCRT) in patients with locally advanced rectal cancer, Table S1: Comparison between pre-treatment clinical factors and response to neoadjuvant chemoradiotherapy (nCRT), considering only patients who received radiotherapy together with capecitabine as chemotherapy (*n* = 157). Odds ratios and 95% confidence intervals are generated from the logistic regression model.
