# Supplementary material for: C-Reactive Protein as Predictive Biomarker for Response to Chemoradiotherapy in Patients with Locally Advanced Rectal Cancer: A Retrospective Study
Source: Cancers (Basel). 2022 Jan 19;14(3):491. doi: 10.3390/cancers14030491 (PMC8833484; doi:10.3390/cancers14030491)
Supplement: Supplementary file 1 [file cancers-14-00491-s001.zip › cancers-1506282-supplementary.pdf]

cancers

MDPI

Supplementary Materials

C-Reactive Protein as Predictive Biomarker for Response to Chemoradiotherapy in Patients with Locally Advanced Rectal Cancer: A Retrospective Study

Fátima Aires, Darlene Rodrigues, María Piñeiro Lamas, Maria Teresa Herdeiro, Adolfo Figueiras , Maria José Oliveira, Margarida Marques and Ana Teresa Pinto

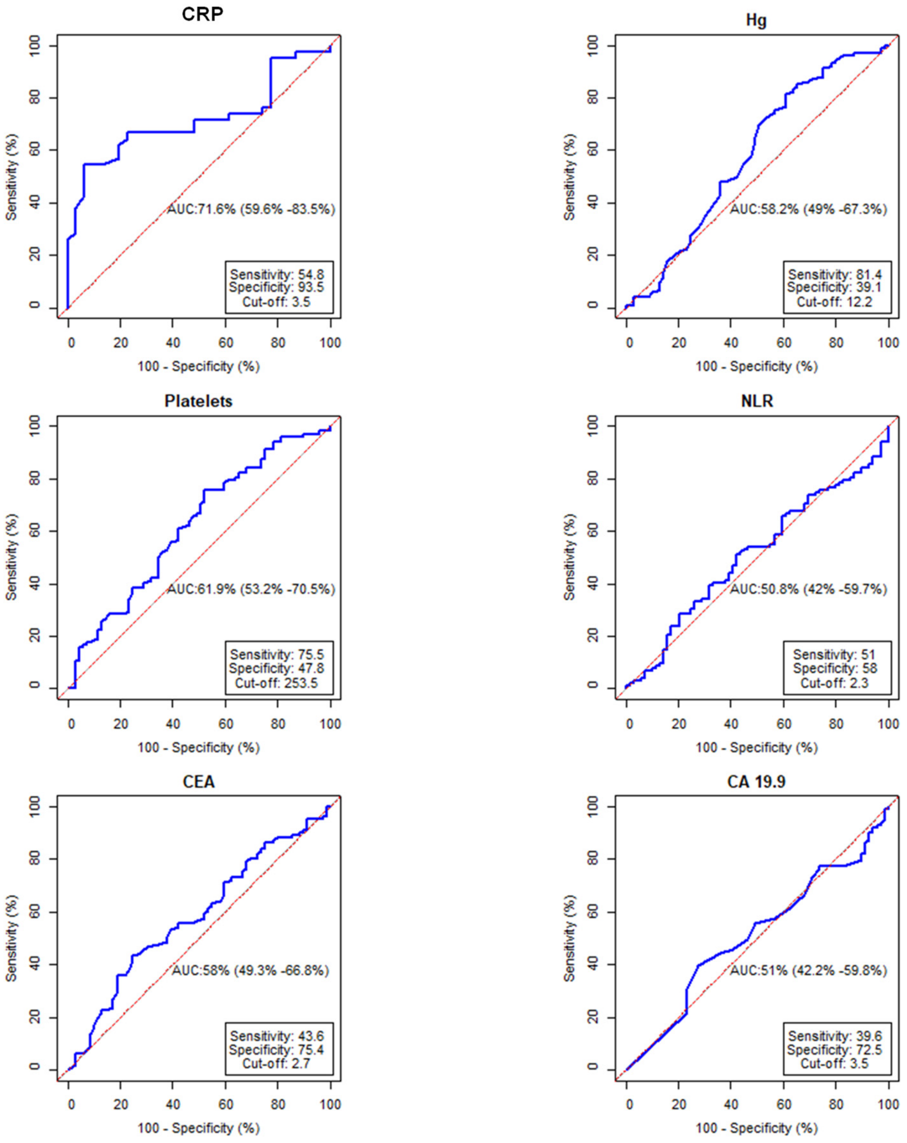

Figure S1. ROC curves for Hg (hemoglobin, g/dl), CRP (C-reactive protein, mg/L), platelets (x10^9/L), NLR (ratio neutrophil lymphocyte), CEA (carcinoembryonic antigen, ng/ml) and CA 19.9 (carbohydrate antigen 19.9, U/ml) levels in patients with nonresponders' group versus responders' group to nCRT. The curves show optimal cut-off value. The plots show optimal cut-off values and the area under the ROC curve (AUC) with the 95% confidence interval.

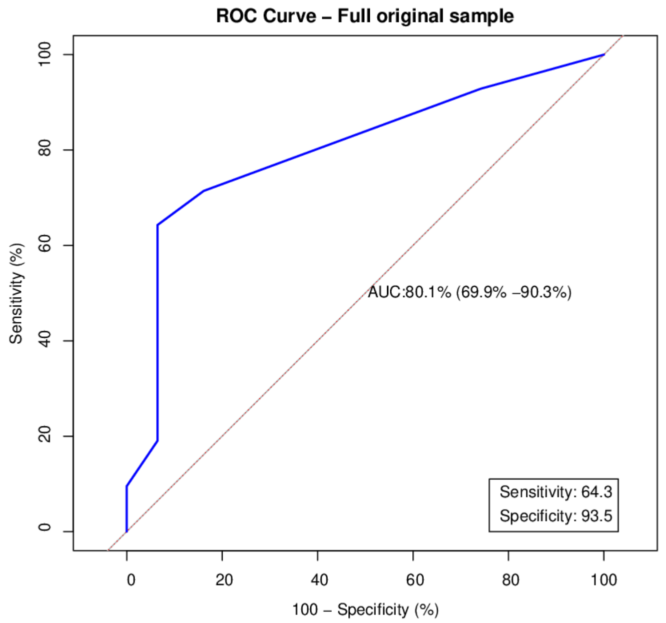

Figure S2. ROC curves for the multivariate regression logistic model fitted on the full sample.

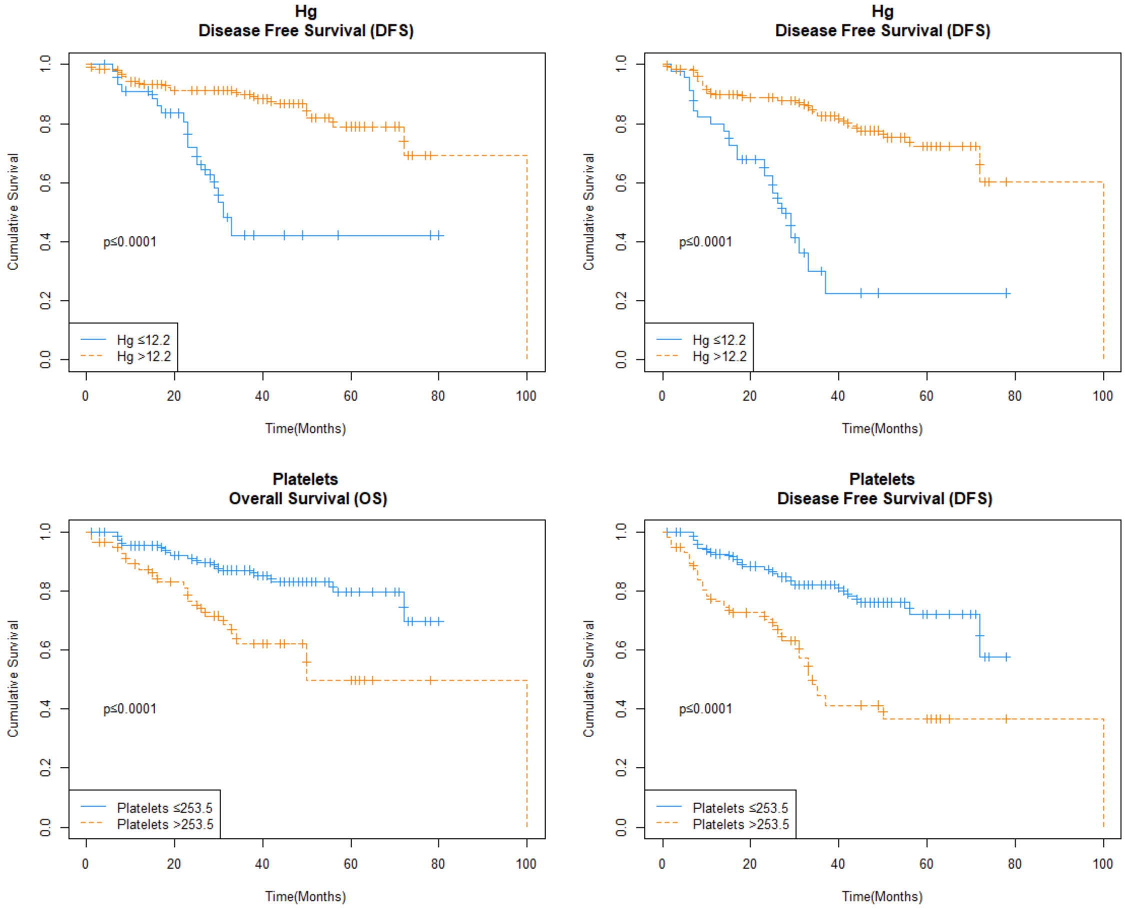

Figure S3. Kaplan–Meier curves for overall survival (OS) and disease-free-survival (DFS) for Hemoglobin (Hg) and Platelets prior to neoadjuvant chemoradiotherapy (nCRT) in patients with locally advanced rectal cancer.

Table S1. Comparison between pre-treatment clinical factors and response to neoadjuvant chemoradiotherapy (nCRT), considering only patients who received radiotherapy together with capecitabine as chemotherapy (*n* = 157). Odds ratios and 95% confidence intervals are generated from the logistic regression model.

Factors	Univariate analysis OR (95% IC)	*p*	Multivariate analysis OR (95% IC)	*p*
cT				
2-3	1 (-)			
4	0.616 (0.272 - 1.4)	0.2433		
ECOG				
0	1 (-)			
1-2	1.041 (0.459 - 2.452)	0.9239		
Hg (g/dl)*				
≤12.2	1 (-)			
>12.2	2.288 (1.068 - 4.969)	0.0339		
CRP (mg/L)*				
≤3.5	1 (-)		1 (-)	
>3.5	0.058 (0.009 - 0.233)	0.0004	0.05 (0.007 - 0.22)	0.0004
Platelets (x10^9/L)*				
≤253.5	1 (-)			
>253.5	0.386 (0.193 - 0.764)	0.0066		
NLR*				
≤2.3	1 (-)		1 (-)	
>2.3	0.736 (0.383 - 1.407)	0.3552	2.151 (0.614 - 8.645)	0.2482
CEA (ng/ml)*				
≤2.7	1 (-)		1 (-)	
>2.7	0.47 (0.228 - 0.937)	0.0352	0.566 (0.122 - 2.458)	0.4473
CA 19.9 (U/ml)*				
≤3.5	1 (-)			
>3.5	0.56 (0.272 - 1.122)	0.1077		

OR: odds ratio; CI: confidence interval; CT: clinical tumor; ECOG: Eastern Cooperative Oncology Group Performance Status; CPR: C-reactive protein; NLR: ratio neutrophil lymphocyte, CEA: carcinoembryonic antigen; CA 19.9: carbohydrate antigen 19.9. * Cut offs – ROC curve analysis
